# Meeting physical activity recommendations is associated with health-related quality of life in women before and after Roux-en-Y gastric bypass surgery

**DOI:** 10.1007/s11136-019-02120-0

**Published:** 2019-02-05

**Authors:** Fanny Sellberg, Sofie Possmark, Mikaela Willmer, Per Tynelius, Margareta Persson, Daniel Berglind

**Affiliations:** 1 Department of Public Health Sciences (PHS), Karolinska Institutet, K9, Social Medicin, 171 77 Stockholm, Sweden; 2 Department of Health and Caring Sciences, University of Gävle, 801 76 Gävle, Sweden; 3 Centre for Epidemiology and Community Medicine, Stockholm County Council, Box 45436, 104 31 Stockholm, Sweden; 4 Department of Nursing, Umeå University, 901 87 Umeå, Sweden

**Keywords:** Health-related quality of life, Physical activity, Bariatric surgery, Gastric bypass, Sedentary time, Step counts

## Abstract

**Purpose:**

Meeting physical activity (PA) recommendations is positively associated with health-related quality of life (HRQoL), but it is still unclear whether PA (specifically objectively measured) is associated with HRQoL in bariatric surgery candidates, both before and after surgery. Thus, the aim of this study was to examine the cross-sectional association between meeting objectively measured PA recommendations and HRQoL before and after Roux-en-Y gastric bypass (RYGB) surgery.

**Methods:**

Sixty-six women undergoing RYGB with pre-surgery and 62 women with post-surgery valid PA and HRQoL data were included from the control group of a RCT study aiming at improving HRQoL and PA post-RYGB surgery. Measures before and 12 months after RYGB included the Short Form Health Survey (SF-36) and objectively measured PA, sedentary time (ST), and step counts with GT3X+ accelerometers. Multiple linear regression models were used to analyze the associations between PA and HRQoL.

**Results:**

Participants who engaged in more than 150 min of moderate to vigorous PA (MVPA)/week (PA recommendations) had considerably higher SF-36 scores (HRQoL) than those who did not, both pre and 12-month post-surgery, with greatest difference in the subscale bodily pain, 15.5 (*p* = 0.021) higher score (higher scores means less pain) pre-surgery and a 19.7 (*p* = 0.004) higher score post-surgery. Higher LPA and step counts and lower ST also showed positive associations in some of the subscales of SF-36.

**Conclusions:**

Meeting the PA recommendations and overall engaging in more PA was associated with higher HRQoL, pre-, and post-RYGB surgery, highlighting the importance of PA both pre- and post-surgery.

**Electronic supplementary material:**

The online version of this article (10.1007/s11136-019-02120-0) contains supplementary material, which is available to authorized users.

## Introduction

Meeting the physical activity (PA) recommendations of at least 150 min of moderate to vigorous PA (MVPA) per week is associated with a wide range of positive health outcomes across all weight ranges, including reduced risk of heart disease, type 2 diabetes, some cancers, and improved mental health [1, 2]. Furthermore, meeting the PA recommendations and higher duration, intensity, and bout length of PA are positively associated with health-related quality of life (HRQoL) [3–5]; a multidimensional measure of physical, mental, functional, and social wellbeing, in the general population [6]. Less is known about sedentary time (ST) and PA associations with HRQoL in individuals suffering from obesity [7, 8] and few studies have used objectively measured PA and ST [9].

Individuals suffering from obesity often show lower levels of HRQoL compared to the general normal weight population [10]. Weight loss has been associated with increases in HRQoL [11], especially rapid weight loss induced by bariatric surgery [12–14], where Roux-en-Y gastric bypass (RYGB) is the most commonly performed bariatric surgery procedure in Sweden [15]. Greatest improvements of HRQoL, induced by weight loss, are often seen on the physical part of the measurement scale, probably caused by the reduced physical complaints after losing weight, but the mental part has also been shown to improve [12, 13].

Peak improvements in HRQoL after bariatric surgery are typically observed during the first years of follow-up, followed by a small decline that usually stabilizes approximately 5 years postoperatively [16, 17]. Although HRQoL improves substantially after bariatric surgery, PA usually does not increase (despite great weight loss) [18–20], except when self-reported PA is used as an outcome measure [20]. The discrepancy between self-reported and objectively measured PA in this patient group is large, and tends to increase post-RYGB [21]. Consequently, objectively measured PA is the preferred method for assessing actual levels of PA in this patient category.

Interventions aiming to increase PA pre-bariatric surgery have been shown to also improve HRQoL pre-surgery [22], but not post-surgery [23]. However, it is still unclear whether objectively measured PA is associated with HRQoL before and after bariatric surgery in candidates receiving regular care (in a non-intervention setting). Thus far, 10-year follow-up data from the Swedish Obese Subjects (SOS) study have shown that HRQoL is improved and associated with weight loss after bariatric surgery. However, the self-reported data on PA, with its inherent bias [24], limit any interpretation on associations between PA and HRQoL before and after RYGB.

The aim of this study was to examine the association between meeting physical activity recommendations, light PA (LPA), ST and step counts (objectively measured), and HRQoL before and 12 months after RYGB surgery. A secondary aim was to explore the association between pattern and intensity of PA and HRQoL changes pre- and 12-month post-RYGB.

## Materials and methods

For the current study, we used the control group of an ongoing randomized controlled intervention, to study the association between PA and HRQoL. The study, named WELL-RYGB, has been described in detail in a previously published protocol paper [25]. In short, the WELL-RYGB is a randomized controlled trial examining the effects of a dissonance-based post-RYGB intervention, on HRQoL and PA at 12- and 24-month follow-up. The study was approved by the regional ethics committee of Stockholm (Dnr:2013/1847–31/2). The trial has also been registered at http://www.isrctn.org with identification number ISRCTN16417174 and all participants have given oral and written consent to participate.

### Participants

Women were recruited from waiting lists for RYGB surgery from five different hospitals in Sweden (Örebro University Hospital, Akademiska Hospital, Ersta Hospital, St. Görans Hospital, and Danderyds Hospital). Inclusion criteria were being eligible for RYGB surgery [body mass index (BMI) > 35 with complications from the obesity or BMI > 40, conducted several serious attempts to lose weight with other methods, and usually over 18 years old] and enough Swedish language skills to be a part of an intervention in Swedish and answer questionnaires in Swedish. Interested patients were sent informed consent forms, a questionnaire measuring HRQoL and demographic characteristics, and an accelerometer before surgery. Participants were included if they returned the informed consent. The same questionnaires and an accelerometer were sent 12 months after surgery, yielding two measure points: baseline (approximately 1 month before RYGB) and 12-month follow-up. We only included the control group in this sub-study since the intervention aimed at improving HRQoL and PA and may therefore, if included in the current study, bias the results. Additionally, there were only women included in the original intervention study because of power concerns. The intervention might affect men and women differently, therefore creating the need for stratification, and it was not possible to collect the double number of participants in order to obtain enough power, especially as approximately 77% of all Swedish RYGB patients are women [15]. All participants had RYGB surgery between April 2015 and June 2017. In the current sub-study, we included 66 women with pre-surgery and 62 women with 12-month post-surgery valid PA and HRQoL data, only 39 women had valid accelerometer data for both measure points.

### HRQoL

HRQoL was measured using the Short Form (36) Health Survey (SF-36). The SF-36 is divided into eight domains: vitality (VT), physical functioning (PF), bodily pain (BP), general health perceptions (GH), physical role functioning (RP), emotional role functioning (RE), social role functioning (SF), and mental health (MH), and can be summarized into two summary scores: physical summary score (PCS) and mental summary score (MCS). Scoring ranges from 0 to 100 with higher scores indicating better quality of life. The instrument performs well in a general population [26] and is commonly used in obese populations [11]. SF-36 was scored using the Quality Metric scoring Software 8.6.12.pptx and the *“*maximum data recovery*”* setting was used for missing values.

### Physical activity

PA, LPA, step counts, and ST were measured objectively with an accelerometer (ActiGraph GT3X+), worn on the right hip. Participants were asked to wear the accelerometer for all waking hours for seven consecutive days. We used vector magnitude activity counts (V_m_), calculated as the square root of the sum of the counts on all three axes, recorded in 10-s epochs and then converted into counts per minutes (cpm). Measurements were accepted as valid if participants had worn the accelerometer for at least 10 h per day during at least 3 days. The number of participants with valid measure at one of the two measure points is reported in Table 2. Wear time, MVPA, and classification of bouts were computed using *ActiLife v.6.13.3*. For wear time, we used an algorithm by Choi et al. [27]. If there were no counts for 60 consecutive minutes or more, with a maximum break of two minutes with non-zero counts, it was classified as non-wear time [28] and consequently removed from analyses. MVPA minutes were calculated as minutes per day in total and also in 10 min bouts. ST was defined as any minute showing < 100 cpm, light physical activity (LPA) was defined as 100–3207 cpm, and MVPA as 3208 cpm or more [29].

### Other variables

Weight and height were objectively measured at the hospitals, in a standardized manner, pre- and 12-month post-surgery and obtained from medical records. Self-reported questionnaire data at baseline and at 1-year follow-up were used to assess current occupation (categorized as working or not working) and level of education (categorized as university level or lower). Long-term sickness was defined by a question asking if participants had a chronic disease, difficulty after an accident, reduced physical function, or long-term health condition. If the respondent answered yes, the following question was asked: does this condition result in reduced work capacity or limit your regular occupation? With response options (1) “not at all,” (2) “yes, to some degree,” and (3) “yes, to a high degree.” BMI was calculated as weight (kg)/ height^2^ (m^2^).

### Statistical analysis

Data in table one and two are presented as means and standard deviation (SD) or in numbers and percentages. Two-tailed *T* tests were used to test for differences in BMI and PA over time. Multiple regression models were used to analyze the associations between LPA and HRQoL, step counts and HRQoL, ST and HRQoL, and between meeting the MVPA recommendations and HRQoL, with MVPA categorized into two groups, more or less than 150 min of MVPA/week. A similar approach was used for MVPA performed in 10-min bouts since the recommendation for PA is at least 150 min of MVPA performed in 10-min bouts (or longer) per week [30]. However, we also analyzed MVPA in non-bouts, since MVPA independent of how it is accumulated, is associated with numerous health benefits [31]. BMI, percent weight loss, and age were not significant confounders and were therefore not included in the adjusted models. Education, occupation, and long-term sickness showed significant confounding effects in several models and were therefore included in all adjusted analyses of MVPA recommendations and HRQoL as well as wear time. For the association between LPA, step counts, ST, and HRQoL, only occupation and long-term sickness were found to be significant confounders and therefore included in the adjusted models, as well as wear time. The majority of the participants only had PA measured at one time-point and there could be systematic differences between those with PA measured at one or two time points. Therefore, sensitivity analyses were performed for the group of participants who had valid PA measures both pre- and post-surgery (*n* = 39) (Supplementary Tables 1 and 2). All statistical analyses were performed with STATA.15.1 (StataCorp).

## Results

Out of the 103 included participants in the study, 90 women had valid accelerometer data for at least one of the measurements, before or 12 months after RYGB. Sixty-six of those had valid accelerometer data and HRQoL data pre-surgery, whereas 62 had valid data on the same measures at 12-month post-RYGB. Thirty-nine women had valid data at both measure points. Characteristics of participants pre- and 12-month post-RYGB are presented in Table 1. Participants with both valid accelerometer measurement had overall slightly higher SF-36 scores pre-surgery, suffered from long-term sickness to a greater extent, and had higher levels of education compared to those with only one valid accelerometer measurement. Mean percentage weight loss at 12-month post-surgery was 47.2% (SD = 16.8) and prevalence of long-term sickness was also reduced to 27% at 12-month post-surgery compared with 59% pre-surgery.

**Table 1:** Participants’ characteristics and health-related quality of life (HRQoL) before and 12 months after Roux-en-Y gastric bypass surgery (RYGB)

Variables	Pre-surgery (*n* = 66)	12-month post-surgery (*n* = 62)	Pre-surgery with valid PA measurements at both time points (*n* = 39)	12-month post-surgery with valid PA measurements at both time points (*n* = 39)
BMI (kg/m^2^)	40.9 (5.3) (*n* = 65)	28.1 (4.2) (*n* = 59)	40.7 (4.3) (*n* = 38)	28.2 (3.9) (*n* = 37)
% weight loss		− 47.2% (16.8)		− 45.7% (15.3)
Age (years)	44.5 (9.7)	47.5 (10.0)	45.8 (9.9)	46.8 (9.9)
Education at university level	21 (32%)	15 (25%)	14 (36%)	13 (33%)
Working	50 (76%)	51 (82%)	31 (79%)	31 (79%)
Suffering from long-term sickness	39 (59%)	17 (27%)	24 (62%)	14 (36%)
Long-term sickness with no limitations	8 (12%)	6 (10%)	8 (21%)	4 (10%)
Long-term sickness with some limitations	18 (27%)	7 (11%)	8 (21%)	6 (15%)
Long-term sickness with a high degree of limitations	13 (20%)	4 (6%)	8 (21%)	4 (10%)
SF-36 subscales				
PF, physical functioning	56.1 (24.1)	89.6 (15.0)	56.5 (25.7)	86.9 (17.8)
RP, physical role functioning	70.6 (30.7)	90.1 (18.5)	73.9 (29.2)	88.8 (19.5)
BP, bodily pain	43.8 (27.6)	75.4 (27.5)	46.0 (26.8)	70.5 (21.8)
GH, general health	50.9 (21.2)	79.1 (19.5)	50.8 (19.4)	77.9 (21.2)
VT, vitality	37.7 (21.3)	64.3 (21.1)	38.6 (20.1)	65.2 (21.9)
SF, social role functioning	62.7 (28.5)	90.7 (19.2)	66.0 (27.6)	90.4 (20.2)
RE, emotional role functioning	76.4 (30.3)	91.4 (18.0)	75.9 (30.6)	90.4 (17.4)
MH, mental health	62.9 (20.9)	81.7 (14.1)	64.1 (19.3)	81.0 (15.5)
PCS, physical summary score	41.5 (9.2)	53.7 (7.6)	42.1 (8.5)	52.5 (8.6)
MCS, mental summary score	45.2 (11.2)	53.5 (7.8)	45.6 (10.1)	53.7 (8.1)

Data presented as mean (SD) or number (percentage)

*BMI* Body mass index, *SF-36* the Short Form (36) Health Survey

**Fig. 1:**
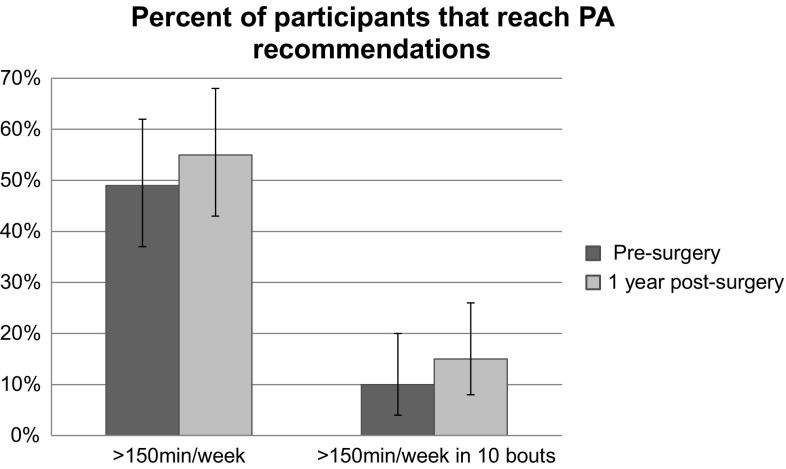
Percentage of participants meeting physical activity recommendations, pre-, and post-RYGB surgery

Table 2 shows participants’ levels of PA, LPA, step counts, and ST pre- and 12-month post-surgery. Mean MVPA min/day pre-surgery were 26.4 (SD = 21.0) and 29.6 (SD = 22.4) min/day 12 months after surgery. Participants also increased their time spent in MVPA in 10-min bouts from 7.5 (SD = 12.6) pre-surgery to 11.6 (SD = 14.6) min/day 12-month post-surgery, although not statistically significant p = 0.334. Step counts increased significantly by 1162.8 steps/day (*p* = 0.014) for those with valid data at one measure point, see Table 2. A similar pattern was found for participants with valid accelerometer PA data at both measure points (data not shown). Ten percent of the participants reached the PA recommendation of ≥ 150 min of MVPA in 10-min bouts per week pre-surgery and 15% at 12-month post-surgery, see Fig. 1.

**Table 2:** Physical activity (PA), sedentary time (ST), and step counts for participants pre-surgery and 12-month post-surgery

Variables	Pre-surgery (*n* = 66)	12-month post-surgery (*n* = 62)	Difference between pre- and post-surgery (*n* = 39)
Mean wear time h/day	14.0 (1.2)	14.8 (1.4)	0.4 (1.6) *p* = 0.089
Mean counts/min	583.9 (229.2)	598.8 (180.0)	6.5 (183.9) p = 0.826
MVPA in 10 bouts, min/day	7.5 (12.6)	11.6 (14.6)	2.7 (16.9) *p* = 0.334
MVPA min/day	26.4 (21.0)	29.6 (22.4)	2.1 (22.6) *p* = 0.566
LPA min/day	359.0 (88.2)	400.7 (78.0)	19.6 (93.3) *p* = 0.197
ST min/day	455.0 (99.3)	457.3 (100.6)	5.1 (94.3) *p* = 0.737
Step counts steps/day	5971.0 (2776.5)	7511.7 (2989.0)	1162.8 (2829.3) *p* = 0.014

Data presented as mean (SD)

*MVPA* moderate to vigorous physical activity, *LPA* light physical activity, *ST* sedentary time

Table 3 shows the mean SF-36 scores comparing participants engaging in more or less than 150 min MVPA/week or more or less than 150 min MVPA in 10 min bouts/week. Participants who engaged in more than 150 min MVPA/week had considerably higher SF-36 scores than those who did not, both pre- and post-surgery. These differences varied greatly for different sub-scores, see Table 3, and most of the found associations decreased or disappeared when adjusting for confounders, in particular long-term sickness. Looking at the summary scores, PCS showed stronger associations with all levels of PA than MCS after surgery but a strong association between LPA and MCS was found pre-surgery with a 2.6-point increase in MCS for each hour increase in LPA, see Table 4. The results in Table 4 are presented as change in SF-36 score per hour increase in LPA or ST and per 1000 increased steps/day.

**Table 3:** Multiple regression analysis: difference in HRQoL by meeting recommendations of either 150 min/week of MVPA in general and in 10 min bouts or not

Variables	MCS	PCS	PF	RP	BP	GH	VT	SF	RE	MH
Pre-surgery (*n* = 66)										
150 min/week MVPA	5.2 (*p* = 0.060)	5.1 (*p* = 0.022)	13.5 (*p* = 0.022)	12.5 (*p* = 0.098)	15.5 (*p* = 0.021)	10.6 (*p* = 0.053)	15.2 (*p* = 0.003)	14.8 (*p* = 0.034)	5.4 (*p* = 0.475)	13.1 (*p* = 0.010)
150 min/week MVPA adjusted^a^	4.0 (*p* = 0.126)	0.3 (*p* = 0.850)	4.7 (*p* = 0.408)	− 2.9 (*p* = 0.585)	3.4 (*p* = 0.571)	3.7 (*p* = 0.445)	9.6 (*p* = 0.040)	10.9 (*p* = 0.123)	− 2.1 (*p* = 0.768)	10.4 (*p* = 0.037)
150 min/week MVPA (10 bouts)	3.5 (*p* = 0.443)	2.4 (*p* = 0.525)	7.5 (*p* = 0.440)	10.9 (*p* = 0.380)	8.8 (*p* = 0.426)	− 1.7 (*p* = 0.843)	6.8 (*p* = 0.431)	13.8 (*p* = 0.229)	3.8 (*p* = 0.759)	7.9 (*p* = 0.346)
150 min/week MVPA (10 bouts) adjusted^a^	4.8 (*p* = 0.232)	1.2 (*p* = 0.650)	7.1 (*p* = 0.408)	9.4 (*p* = 0.246)	6.2 (*p* = 0.502)	− 3.8 (*p* = 0.598)	8.2 (*p* = 0.258)	15.8 (*p* = 0.142)	4.5 (*p* = 0.677)	10.1 (*p* = 0.190)
12-month post-surgery (*n* = 62)										
150 min/week MVPA	0.6 (*p* = 0.758)	6.9 (*p* < 0.001)	15.4 (*p* < 0.001)	10.4 (*p* = 0.027)	19.7 (*p* = 0.004)	10.1 (*p* = 0.042)	5.3 (*p* = 0.334)	10.6 (*p* = 0.029)	6.6 (*p* = 0.153)	1.4 (*p* = 0.709)
150 min/week MVPA adjusted^a^	− 1.7 (*p* = 0.375)	4.0 (*p* = 0.003)	10.2 (*p* = 0.001)	3.7 (*p* = 0.278)	10.0 (*p* = 0.088)	2.4 (*p* = 0.480)	1.3 (*p* = 0.789)	4.0 (*p* = 0.355)	0.0 (*p* = 0.998)	− 3.1 (*p* = 0.368)
150 min/week MVPA (10 bouts)	3.1 (*p* = 0.255)	7.1 (*p* = 0.006)	12.4 (*p* = 0.015)	10.3 (*p* = 0.107)	23.6 (*p* = 0.012)	15.2 (*p* = 0.022)	16.5 (*p* = 0.022)	9.6 (*p* = 0.150)	8.3 (*p* = 0.187)	5.1 (*p* = 0.294)
150 min/week MVPA (10 bouts) adjusted^a^	− 0.5 (*p* = 0.841)	3.1 (*p* = 0.096)	5.8 (*p* = 0.166)	0.5 (*p* = 0.917)	9.7 (*p* = 0.214)	3.5 (*p* = 0.446)	9.5 (*p* = 0.125)	− 0.2 (*p* = 0.971)	− 1.6 (*p* = 0.770)	− 1.2 (*p* = 0.785)

Data presented as β (p-value)

*MVPA* moderate to vigorous physical activity, *PF* physical functioning, *RP* physical role functioning, *BP* bodily pain, *GH* general health perceptions, *VT* vitality, *SF* social role functioning, *RE* emotional role functioning, *MH* mental health, *PCS* physical summary score, *MCS* mental summary score

^a^Adjusted for occupation, education, wear time, and long-term sickness

**Table 4:** Multiple regression analysis: linear association between HRQoL and light physical activity (LPA, per 60 min), sedentary time (ST, per 60 min), or step counts (per 1000 steps)

Variables	MCS	PCS	PF	RP	BP	GH	VT	SF	RE	MH
Pre-surgery (*n* = 66)										
LPA	2.6 (*p* = 0.005)	1.7 (*p* = 0.030)	4.1 (*p* = 0.041)	3.4 (*p* = 0.193)	5.6 (*p* = 0.016)	5.0 (*p* = 0.004)	6.1 (*p* < 0.001)	9.2 (*p* < 0.001)	2.4 (*p* = 0.362)	5.1 (*p* = 0.003)
LPA adjusted^a^	1.7 (*p* = 0.055)	0.3 (*p* = 0.565)	1.6 (*p* = 0.399)	− 2.2 (*p* = 0.211)	2.5 (*p* = 0.230)	2.7 (*p* = 0.087)	3.6 (*p* = 0.019)	7.2 (*p* = 0.002)	− 1.2 (*p* = 0.603)	3.5 (*p* = 0.037)
ST	− 1.1 (*p* = 0.171)	− 0.6 (*p* = 0.414)	− 1.4 (*p* = 0.436)	− 0.0 (*p* = 0.990)	− 2.4 (*p* = 0.253)	− 1.8 (*p* = 0.270)	− 3.7 (*p* = 0.021)	− 4.7 (*p* = 0.026)	− 0.6 (*p* = 0.806)	− 1.4 (*p* = 0.365)
ST adjusted^a^	− 1.0 (*p* = 0.191)	− 0.0 (*p* = 0.999)	− 0.5 (*p* = 0.753)	2.0 (*p* = 0.193)	− 1.2 (*p* = 0.495)	− 0.7 (*p* = 0.596)	− 2.9 (*p* = 0.029)	− 4.1 (*p* = 0.040)	0.4 (*p* = 0.856)	− 1.2 (*p* = 0.424)
Step counts	1.2 (*p* = 0.015)	1.3 (*p* = 0.001)	2.9 (*p* = 0.006)	4.1 (*p* = 0.002)	3.4 (*p* = 0.006)	2.7 (*p* = 0.003)	2.8 (*p* = 0.002)	4.0 (*p* = 0.001)	2.5 (*p* = 0.070)	2.4 (*p* = 0.010)
Step counts adjusted^a^	0.7 (*p* = 0.166)	0.4 (*p* = 0.271)	1.2 (*p* = 0.244)	0.5 (*p* = 0.604)	1.3 (*p* = 0.268)	1.2 (*p* = 0.190)	1.1 (*p* = 0.198)	2.7 (*p* = 0.038)	0.4 (*p* = 0.786)	1.5 (*p* = 0.104)
12-month post-surgery (*n* = 62)										
LPA	1.3 (*p* = 0.097)	1.5 (*p* = 0.041)	2.6 (*p* = 0.081)	3.9 (*p* = 0.029)	3.9 (*p* = 0.148)	3.8 (*p* = 0.045)	5.0 (*p* = 0.015)	3.8 (*p* = 0.045)	3.2 (*p* = 0.069)	1.2 (*p* = 0.375)
LPA adjusted^a^	0.5 (*p* = 0.538)	− 0.1 (*p* = 0.851)	− 0.2 (*p* = 0.838)	0.4 (*p* = 0.753)	− 1.2 (*p* = 0.587)	0.1 (*p* = 0.970)	2.7 (*p* = 0.130)	1.1 (*p* = 0.522)	0.6 (*p* = 0.714)	− 0.5 (*p* = 0.719)
ST	− 0.6 (*p* = 0.332)	− 1.3 (*p* = 0.024)	− 1.6 (*p* = 0.169)	− 3.3 (*p* = 0.017)	− 3.7 (*p* = 0.076)	− 2.3 (*p* = 0.127)	− 3.5 (*p* = 0.030)	− 2.8 (*p* = 0.054)	− 2.2 (*p* = 0.113)	0.2 (*p* = 0.842)
ST adjusted^a^	− 0.1 (*p* = 0.788)	− 0.8 (*p* = 0.055)	− 1.1 (*p* = 0.268)	− 2.1 (*p* = 0.025)	− 1.9 (*p* = 0.281)	− 0.5 (*p* = 0.619)	− 1.9 (*p* = 0.155)	− 2.0 (*p* = 0.114)	− 1.3 (*p* = 0.270)	1.0 (*p* = 0.312)
Step counts	0.2 (*p* = 0.546)	1.3 (*p* < 0.001)	2.5 (*p* < 0.001)	2.6 (*p* = 0.001)	3.6 (*p* = 0.002)	2.2 (*p* = 0.010)	1.4 (*p* = 0.128)	1.5 (*p* = 0.071)	1.9 (*p* = 0.019)	0.2 (*p* = 0.775)
Step counts adjusted^a^	− 0.4 (*p* = 0.277)	0.6 (*p* = 0.013)	1.3 (*p* = 0.023)	0.5 (*p* = 0.457)	1.5 (*p* = 0.182)	0.4 (*p* = 0.526)	0.3 (*p* = 0.731)	0.3 (*p* = 0.742)	0.0 (*p* = 0.993)	− 1.0 (*p* = 0.120)

Data presented as β (*p* value)

*LPA* light physical activity, *ST* sedentary time, *PF* Physical functioning, *RP* Physical role functioning, *BP* Bodily pain, *GH* General health perceptions, *VT* vitality, *SF* Social role functioning, *RE* Emotional role functioning, *MH* Mental health, *PCS* Physical summary score, *MCS* Mental summary score

^a^Adjusted for education, wear time, and long-term sickness

### Sensitivity analyses

Sensitivity analyses of mean scores of SF-36 comparing more or less than 150 min/week of MVPA and more or less than 150 min of MVPA in 10 min bouts/week for participants with valid PA measures at both measure points can be found in Supplementary Table 1 (*n* = 39). In general, there were slightly weaker associations pre-surgery, and in almost all subscales the associations 12-month post-surgery were stronger, with bodily pain showing very strong association engaging in ≥ 150 min MVPA/week, in average 29.1 (*p* = 0.001) higher score in bodily pain (i.e., meaning less pain) compared to those who did not; same numbers for engaging in ≥ 150 min MVPA/week in 10 min bouts were 29.5 (*p* = 0.008). Supplementary Table 2 shows associations between LPA, ST, step counts, and HRQoL for participants with valid PA measures at both measure points; the table shows similar results to the analyses including all participants.

Additionally, we also checked for if changes in physical activity (going from active, meeting PA recommendations, to inactive, not meeting PA recommendations, or the other way around) were associated with changes in HRQoL in these 39 participants. Unfortunately, there were too few participants to see any valid results, see Supplementary Material 3. However, we saw no relevant differences in changes in HRQoL between participants being active before surgery compared to inactive (mean improvement in MCS = 7.4 (SD = 8.3) vs. 8.7 (SD = 9.7) and for PCS = 9.7 (SD = 6.6) vs. 11.1 (SD = 8.6), respectively).

Moreover, we run the same analyses as above including all participants with accelerometer data (without any criteria of wear time). This only added two participants and did not change any of the results substantially.

## Discussion

Meeting the PA recommendations of ≥ 150 min of MVPA per week in non-bouts and in 10-min bouts was associated with higher HRQoL summary scores as well as in many of the subscales pre- and post-RYGB and associations were found to be stronger post-surgery. Additionally, we also found associations for LPA, ST, and step counts with HRQoL pre- and post-RYGB. However, most of the associations were decreased or diminished when adjusting for relevant confounders, especially when adjusting for living with long-term sickness. In general, participating women improved their HRQoL substantially from pre- to 12-month post-RYGB, especially on subscales connected to the physical part of HRQoL.

Pre-surgery, women’s HRQoL scores were considerably lower than among the general population [32]. However, at 12-month post-RYGB, scores were similar, and in some subscales even higher, than among the general population [32]. All intensity levels of PA were higher 12-month post-surgery compared to pre-surgery, although not statistically significant, except for step counts. Great improvements in HRQoL after bariatric surgery, in line with our results, have been seen in several previous studies [12, 13, 23], with a peak improvement at 12-month post-surgery [33]. Contrary to previous studies on objectively measured PA after bariatric surgery [34, 35], participants in the current study increased both MVPA and LPA at 12-month post-surgery. However, this increase was quite small and statistically non-significant. Pre-surgery, approximately 50% meet the recommended levels of ≥ 150 min of MVPA per week, compared with 55% 12-month post-surgery. On the other hand, only 10% pre- and 15% 12-month post-surgery met the recommended levels of ≥ 150 min of MVPA per week performed in 10-min bouts, as stated in the PA recommendations [30]. These proportions of participants meeting the PA recommendations are similar to those reported in a previous study with objectively measured PA in women undergoing RYGB [36], with the exception of fewer women meeting 150 min MVPA per week pre-surgery in the current study.

As stated before, the general recommendation for PA for the age group 17–64 is at least 150 min of weekly MVPA performed in bouts of at least 10 min [30]. We chose to additionally look at 150 min of MVPA per week in non-bouts, since few participants met the PA recommendation and due to the fact that recently published data show that MVPA, independent of how it is accumulated throughout the day, is associated with numerous health benefits [31]. Although meeting the recommended levels of PA is an appropriate long-term goal for most RYGB patients, this may be too challenging for many patients. Thus, setting realistic, achievable, assessable short-term goals (e.g., 150 min MVPA per week in non-bouts), and gradually increasing the amount and intensity of PA over time may be a conceivable strategy [37].

In general, the associations between meeting the PA recommendations of ≥ 150 min of MVPA per week and higher HRQoL tended to be stronger at 12-month post-RYGB compared to pre-surgery, both for general HRQoL scores and for the different subscales. Both levels of MVPA and scores for the subscales connected to the physical part of HRQoL increased 12-month post-surgery. This could be due to the importance of physical health among severely obese individuals, where for example, bodily pain was scored much higher (high scores indicate less pain) for participants that were able to and were active ≥ 150 min of MVPA per week. When taking into account long-term sickness, the association between meeting the PA recommendations and HRQoL became weaker, supporting this theory. For the subgroup of 39 women who had accelerometer data at both measurement points, the association between meeting PA recommendations and HRQoL was somewhat stronger. They also had a somewhat higher prevalence of long-term sickness, which is in line with the theory above. However, the mean levels of PA in this patient group did not increase post-surgery. Thus, the slightly increased association might also be a function of weight loss, although this is only speculative. The strong associations seen between meeting the 150 min of MVPA per week recommendations and SF-36 were also seen for LPA, step counts, and ST, although difficult to compare since they were analyzed as continuous outcomes. However, increasing LPA with 1 h or increasing with 1000 steps per day might be preferred for some patients. Thus, an increase in both MVPA, LPA, and step counts could be recommended for this patient group.

A previous PA intervention study with objectively measured PA, in 75 pre- bariatric surgery patients, showed that patients who reached larger increases in MVPA also displayed greater improvements in HRQoL, especially for the physical health subscales (physical function, bodily pain, general health, and overall physical health) regardless of age, degree of obesity, and initial baseline levels of MVPA and HRQoL [38]. A similar study also found higher levels of some parts of HRQoL (general health and role physical) after a 24-week physical training intervention compared to a control group [23]. Another study assessing associations between PA and HRQoL before and after bariatric surgery found that patients who were inactive before and became active after surgery and those who were active before and after surgery reported greater improvements in HRQoL, especially in the physical health scores, compared with patients who were active before and became inactive after surgery and those who were inactive both before and after surgery [39]. However, those findings have limited comparability with results from the current study since the study used self-reported data on PA which has shown large discrepancies compared to objective measures of PA, which seem to be even greater after, compared to before, bariatric surgery [24]. Future research should consider intervention settings where levels of PA are manipulated at pre- and post-RYGB (and measured objectively) to study potential effects on HRQoL.

### Limitations

The results of the current study should be interpreted in light of its limitations. The inclusion criteria used to assess participants’ eligibility to enroll in the study purposefully excluded those with < 10 h of daily accelerometer wear time data for <3 days; this was not a problem since including those two participants with less accelerometer data than 10 h at least 3 days did not change the results. Additionally, participants in these analyses arise from RYGB patients that wanted to participate in a RCT improving HRQoL and PA after surgery and may therefore have influenced the findings of the study. We cannot tell whether increased PA results in better HRQoL or if better HRQoL makes people more active, since the analyses are cross-sectional and we lacked statistical power to do longitudinal analyses. However, a previous study on obese individuals waiting for RYGB surgery showed greater improvements in HRQoL in an intervention group who increased their PA compared to a control group who did not increase their levels of PA [22]. Secondly, we adjusted for long-term sickness which could act as a moderator, rather than a confounder. However, we still believe that long-term sickness can affect PA levels and HRQoL and therefore can be seen as a confounder. Thirdly, the majority of participants only had valid accelerometer data at one measure point and there could be systematic differences in descriptive and outcome variables between these participants and those who had valid PA data at both measure points. However, we conducted sensitivity analyses using this subgroup and there were indeed differences both in the baseline characteristics and the association between PA, step counts, ST, and SF-36 scores, although this could be due to unstable results because of the small sample size in the sensitivity analyses (*n* = 39). At baseline, long-term sickness was more prevalent; education levels were higher; and HRQoL scores were slightly higher among those with both valid PA measurement points. Fourth, one potential limitation from using objectively accelerometer measured PA and ST is that the method is not able to specify specific domains or types of PA and ST (e.g., occupational/transportation PA/ST or sitting vs. standing still). A final potential limitation related to the lack of consensus on how to define intensities of PA via accelerometer data intensity cut-offs. This limits the comparability with other studies having used different cut-offs to define PA intensities.

### Strengths

This study also shows several strengths. The main strength is the objectively measured PA and ST in 66 obese individuals pre-RYGB and 62 individuals at 12-month follow-up. Another strength is that we were able to examine 12-month post-surgery data, i.e., a period when most RYGB patients have recovered well enough from the surgery to be active again. However, a longer follow-up than 12 month would be of interest since it is known that HRQoL decreases again 1–2 years post-surgery [33]. Further strengths include the objective height and weight measures which were made in a standardized manner, in a hospital setting, both pre- and post-RYGB, which ensures the accuracy of weight loss data.

## Conclusions

The current study found a strong association between meeting guideline PA levels of at least 150 min of MVPA per week and higher HRQoL both pre- and 12-month post-RYGB surgery. Similar results were found for LPA, ST, and step counts both pre- and post-surgery. There were overall higher scores on subscales measuring the physical part of HRQoL, compared to mental parts (both with strongest association for bodily pain and vitality and no relevant associations for the subscale measuring emotional role functioning). These results raise a hypothesis of a possible effect of PA, especially meeting the recommendations of ≥ 150 min MVPA per week either in non-bouts or in 10-min bouts, for obese individuals in general but also after RYGB surgery to improve and keep a higher quality of life.

## Electronic supplementary material

Below is the link to the electronic supplementary material.

Supplementary material 1 (PDF 154 KB)
