# Supplementary material for: Meeting physical activity recommendations is associated with health-related quality of life in women before and after Roux-en-Y gastric bypass surgery
Source: Qual Life Res. 2019 Feb 5;28(6):1497–507. doi: 10.1007/s11136-019-02120-0 (PMC6522440; doi:10.1007/s11136-019-02120-0)
Supplement: Supplementary file 1 — Supplementary material 1 (PDF 154 KB) [file 11136_2019_2120_MOESM1_ESM.pdf]

Supplementary material for article titled: *Meeting physical activity recommendations is associated with health-related quality of life in women before and after Roux-en-Y Gastric Bypass surgery*

Journal: Quality of Life Research

Authors: Fanny Sellberg, Sofie Possmark, Mikaela Willmer, Per Tynelius, Margareta Persson, Daniel Berglind

Corresponding author: Fanny Sellberg, fanny.sellberg@ki.se. Department of Public Health Sciences (PHS), Karolinska Institutet,

Supplementary material 1. Multiple regression analysis: difference in HRQoL by meeting recommendations of either 150 min/week of MVPA in general and in 10 min bouts or not for 39 participants with valid measurements pre- and 12 months post-surgery.

Variables	MCS	PCS	PF	RP	BP	GH	VT	SF	RE	MH
Pre-surgery (n=39)										
150min/week MVPA	2.9 (p=0.378)	4.5 (p=0.099)	15.8 (p=0.055)	10.8 (p=0.254)	10.9 (p=0.211)	6.2 (p=0.325)	6.6 (p=0.312)	9.1 (p=0.310)	4.3 (p=0.671)	10.7 (p=0.083)
150min/week MVPA Adjusted[1]	-0.5 (p=0.869)	-1.0 (p=0.680)	2.7 (p=0.739)	-2.5 (p=0.747)	-5.0 (p=0.550)	-3.3 (p=0.588)	-2.6 (p=0.616)	-2.6 (p=0.785)	-4.8 (p=0.627)	3.6 (p=0.562)
150min/week MVPA (10 bouts)	1.7 (p=0.712)	-0.0 (p=0.993)	1.4 (p=0.922)	3.8 (p=0.774)	4.9 (p=0.687)	-6.1 (p=0.484)	1.1 (p=0.900)	5.7 (p=0.649)	0.6 (p=0.965)	5.0 (p=0.566)
150min/week MVPA (10 bouts) Adjusted[1]	4.1 (p=0.297)	-1.2 (p=0.692)	-0.3 (p=0.977)	4.9 (p=0.617)	1.7 (p=0.871)	-6.7 (p=0.388)	6.7 (p=0.310)	7.3 (p=0.554)	4.2 (p=0.738)	7.8 (p=0.320)
1 year post-surgery (n=39)										
150min/week MVPA	0.4 (p=0.896)	10.2 (p<0.001)	23.9 (p<0.001)	15.4 (p=0.013)	29.1 (p=0.001)	13.5 (p=0.049)	4.0 (p=0.584)	20.5 (p=0.003)	9.3 (p=0.100)	1.7 (p=0.736)
150min/week MVPA Adjusted[1]	-3.5 (p=0.190)	6.4 (p=0.001)	17.4 (p<0.001)	7.0 (p=0.112)	14.7 (p=0.060)	1.7 (p=0.715)	-3.3 (p=0.547)	7.2 (p=0.5245)	0.2 (p=0.968)	-5.8 (p=0.234)
150min/week MVPA (10 bouts)	2.1 (p=0.519)	9.0 (p=0.006)	16.4 (p=0.018)	12.1 (p=0.117)	29.5 (p=0.008)	16.8 (p=0.009)	15.2 (p=0.078)	10.1 (p=0.209)	9.5 (p=0.172)	3.4 (p=0.585)
150min/week MVPA (10 bouts) Adjusted[1]	-3.4 (p=0.256)	3.4 (p=0.140)	5.4 (p=0.340)	-0.7 (p=0.895)	11.7 (p=0.200)	0.1 (p=0.983)	1.2 (p=0.853)	-2.6 (p=0.719)	-2.1 (p=0.717)	-7.9 (p=0.155)

Data presented as β (p-value). MVPA: Moderate to vigorous physical activity, PF: Physical functioning, RP: Physical role functioning, BP: Bodily pain, GH: General health perceptions, VT: vitality, SF: Social role functioning, RE: Emotional role functioning, MH: Mental health, PCS: Physical summary score, MCS: Mental summary score.

[1]Adjusted for occupation, education, wear time and long term sickness.

Supplementary material 2. Multiple regression analysis: linear association between HRQoL and light physical activity (LPA, per 60 min), sedentary time (ST, per 60 min) or step counts (per 1000 steps) for 39 participants with valid measurements pre- and 12 months post-surgery.

Variables	MCS	PCS	PF	RP	BP	GH	VT	SF	RE	MH
Pre-surgery (n=39)										
LPA	1.7 (p=0.216)	1.6 (p=0.156)	3.3 (p=0.334)	1.8 (p=0.655)	4.8 (p=0.176)	5.5 (p=0.028)	4.7 (p=0.073)	6.2 (p=0.088)	0.9 (p=0.822)	3.5 (p=0.170)
LPA Adjusted[1]	-0.4 (p=0.774)	-0.1 (p=0.905)	-0.7 (p=0.839)	-5.3 (p=0.078)	1.3 (p=0.743)	1.3 (p=0.605)	0.3 (p=0.887)	1.3 (p=0.742)	-4.8 (p=0.216)	0.3 (p=0.896)
ST	0.3 (p=0.817)	-0.1 (p=0.934)	1.0 (p=0.714)	1.7 (p=0.595)	-1.3 (p=0.651)	-0.7 (p=0.731)	-1.0 (p=0.656)	0.5 (p=0.869)	0.8 (p=0.813)	1.1 (p=0.604)
ST Adjusted[1]	0.1 (p=0.913)	0.1 (p=0.939)	0.1 (p=0.968)	3.9 (p=0.149)	-1.2 (p=0.729)	-0.8 (p=0.723)	-0.6 (p=0.772)	-1.1 (p=0.754)	3.6 (p=0.305)	-0.8 (p=0.721)
Step counts	0.6 (p=0.293)	0.9 (p=0.077)	2.1 (p=0.169)	3.0 (p=0.077)	2.0 (p=0.207)	1.4 (p=0.208)	1.9 (p=0.108)	1.6 (p=0.313)	2.0 (p=0.275)	1.1 (p=0.326)
Step counts Adjusted[1]	-0.1 (p=0.797)	0.1 (p=0.818)	0.3 (p=0.850)	-0.0 (p=0.985)	0.3 (p=0.840)	-0.1 (p=0.917)	-0.0 (p=0.967)	-0.3 (p=0.837)	-0.2 (p=0.885)	-0.1 (p=0.932)
1 year post-surgery (n=39)										
LPA	1.5 (p=0.139)	2.1 (p=0.043)	3.7 (p=0.083)	4.8 (p=0.038)	5.5 (p=0.119)	4.9 (p=0.057)	6.5 (p=0.012)	4.3 (p=0.082)	2.6 (p=0.231)	2.4 (p=0.200)
LPA Adjusted[1]	0.1 (p=0.896)	1.1 (p=0.150)	2.2 (p=0.249)	3.2 (p=0.055)	1.7 (p=0.591)	1.0 (p=0.595)	3.4 (p=0.097)	1.2 (p=0.616)	0.0 (p=0.984)	0.1 (p=0.953)
ST	0.2 (p=0.853)	-2.2 (p=0.023)	-3.7 (p=0.070)	-3.9 (p=0.077)	-5.7 (p=0.086)	-2.9 (p=0.240)	-3.1 (p=0.223)	-2.1 (p=0.383)	0.1 (p=0.957)	-0.1 (p=0.963)
ST Adjusted[1]	0.3 (p=0.760)	-1.4 (p=0.037)	-3.0 (p=0.081)	-2.8 (p=0.063)	-3.1 (p=0.289)	-1.0 (p=0.548)	-2.2 (p=0.227)	-1.4 (p=0.523)	0.1 (p=0.961)	0.6 (p=0.726)
Step counts	0.2 (p=0.662)	1.6 (p<0.001)	3.5 (p<0.001)	2.8 (p=0.003)	4.6 (p=0.001)	2.4 (p=0.029)	1.6 (p=0.149)	1.9 (p=0.064)	1.7 (p=0.050)	0.4 (p=0.585)
Step counts Adjusted[1]	-0.7 (p=0.073)	0.9 (p=0.003)	2.3 (p=0.003)	0.5 (p=0.445)	2.4 (p=0.073)	0.0 (p=0.972)	-0.4 (p=0.629)	0.3 (p=0.775)	-0.5 (p=0.526)	-1.2 (p=0.108)

Data presented as β (p-value). MVPA: Moderate to vigorous physical activity, PF: Physical functioning, RP: Physical role functioning, BP: Bodily pain, GH: General health perceptions, VT: vitality, SF: Social role functioning, RE: Emotional role functioning, MH: Mental health, PCS: Physical summary score, MCS: Mental summary score.

[1]Adjusted for education, wear time and long term sickness.

Supplementary material 3. Number of participants reaching the PA recommendations (150 min MVPA/ week) pre- and post-surgery.

		Post-surgery	
		active	inactive
Pre-surgery	active	15	6
	inactive	8	11
